# Ultrasound-Guided Glucocorticoid Injection as a Treatment for Rotator Cuff Calcific Tendinopathy: A Case Report

**DOI:** 10.7759/cureus.68934

**Published:** 2024-09-08

**Authors:** Noah E Sobel, Wei Li, Jordyn Williams

**Affiliations:** 1 Department of Emergency Medicine, University of Connecticut School of Medicine, Farmington, USA; 2 Department of Sports Medicine, Hartford HealthCare Medical Group, Bristol, USA; 3 Department of Family Medicine, Hartford HealthCare Medical Group, Bristol, USA; 4 Department of Surgery, University of Connecticut School of Medicine, Farmington, USA

**Keywords:** calcific tendinopathy, corticosteroid injection, non-surgical orthopedics, rotator cuff pathology, ultrasound-guided injection

## Abstract

Rotator cuff calcific tendinopathy (RCCT) is a common disorder of the rotator cuff causing shoulder pain and dysfunction. RCCT is characterized by calcium deposition on and around the tendons of the rotator cuff muscles. Treatment is typically conservative, consisting of anti-inflammatory drugs (NSAIDs) and physical therapy, although certain patients require more invasive treatment. If first-line treatments do not resolve the pain, second-line treatments such as glucocorticoid injections, extracorporeal shock wave therapy (ESWT), barbotage, and surgery may be considered; however, there is no gold standard treatment for these refractory cases. In this case study, a 36-year-old female patient with confirmed RCCT achieved symptom remission with ultrasound-guided methylprednisolone injection followed by adjunctive physical therapy. Ultrasonography enabled precise, targeted delivery of steroids to the calcified lesions, with near 100% resolution of deposits on repeat radiography. With additional physical therapy, the patient was completely pain-free with a full range of motion and the ability to perform daily activities. This case report demonstrates that ultrasound-guided glucocorticoid injection can be an efficacious treatment option for refractory cases of RCCT.

## Introduction

Shoulder pain is a frequent concern in sports medicine and primary care clinics, with the most common source being rotator cuff pathology [1]. Rotator cuff dysfunction or injury can lead to symptoms of shoulder pain and limitation, especially with abduction and internal and external rotation of the shoulder. Without proper diagnosis and treatment, rotator cuff pathology can cause severe pain and major impairment in exercise and daily activities. Rotator cuff calcific tendinopathy (RCCT), characterized by calcium deposition onto rotator cuff tendons, is a frequent cause of debilitating shoulder injury, occurring in 3-10% of the general population. 70% of cases occur in women, often in their 4th and 5th decade of life, and an estimated 20% of patients have RCCT but remain symptomatic [2]. Notably, RCCT is found in up to 17% of patients who are being evaluated for shoulder pain [3]. The most common site of calcium deposition is the supraspinatus tendon (80% of cases) which can lead to subacromial impingement [1].

Management and treatment of RCCT can include both conservative and invasive modalities. Conservative management, targeted at the resolution of pain, involves the use of nonsteroidal anti-inflammatory drugs (NSAIDs), corticosteroid injections, and physical therapy. Patients with poor prognostic factors (bilateral disease, anterior acromion involvement, subacromial extension, and deposits ≥1500 mm^3^) often require invasive treatment options for symptom resolution and calcium deposit regression [4].

Treatment of refractory cases of RCCT is variable as there are no universal clinical guidelines for the management of chronic disease [3]. Refractory RCCT is often managed with glucocorticoid injections, usually administered by palpation-guided or landmark-guided injection into the intra-articular or subacromial space. Extracorporeal shock wave therapy (ESWT), a method of disrupting calcium deposits using acoustic waves, is a treatment that has been used since the 1990s but has inconsistent results in clinical trials [5,6].

Cases of RCCT resistant to nonsurgical management require surgical intervention (approximately 10% of patients) in the form of arthroscopic acromioplasty or open calcium deposit removal [3]. Surgical intervention, and the preceding advanced diagnostic imaging, come with potential risks and complications such as adhesive capsulitis or prolonged shoulder stiffness [3]. Our case demonstrates the use of ultrasound-guided targeted corticosteroid injection for RCCT, an option that obviates the need for advanced imaging and avoids the costs and risks of surgery.

## Case presentation

**Figure 1:**
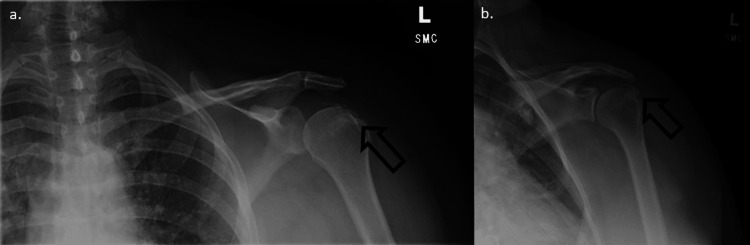
Radiographs of the left shoulder demonstrating rotator cuff calcific tendinitis. a) Anterior-posterior view. b) Internal rotation view.

**Figure 2:**
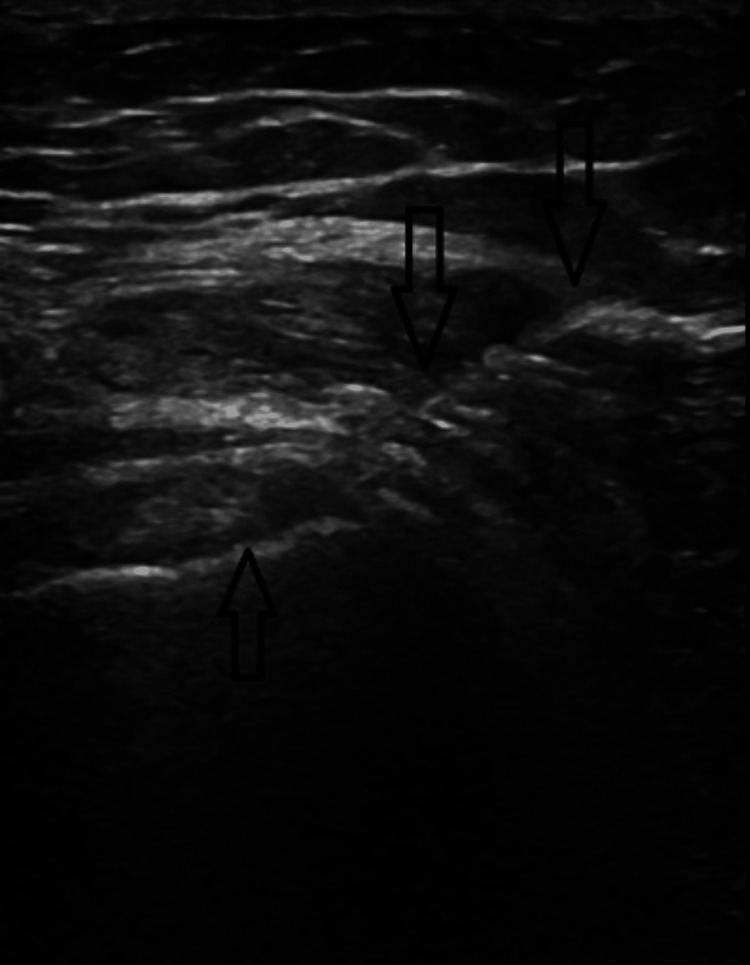
Snapshot of the ultrasound-guided methylprednisolone injection targeting calcific deposits on the supraspinatus tendon and the surrounding area, with arrows indicating the path of the needle.

Our patient is a 36-year-old female seen for a chief complaint of acute left shoulder pain. She presented for a follow-up for persistent and severe pain after receiving a diagnosis of calcific tendinitis in the ER. On the initial exam, she had greatly reduced passive and active range of motion (ROM) with abduction and external rotation, marked tenderness of the supraspinatus, and positive Empty can, Hawkins, and Neer special tests. Initial differential diagnoses included rotator cuff dysfunction or tear, subacromial impingement, adhesive capsulitis, and calcific tendinopathy. Radiographs of the left shoulder were obtained to confirm the diagnosis of rotator cuff calcific tendinitis (Figure 1). Extensive calcific deposits along the supraspinatus tendon were visualized. Based on prior experience with RCCT and a preference for precision, we decided that the best course of treatment was an ultrasound-guided glucocorticoid (methylprednisolone) injection targeting the bursa surrounding the calcium deposits along the supraspinatus tendon (Figure 2). We also recommended that the patient start physical therapy two or three times weekly for her shoulder.

The patient was seen four weeks later for follow-up. She described doing extremely well with significant improvement in her baseline symptoms and pain. She now only had mild pain with passive and active range of motion, largely at the end range. On examination, there was minimal tenderness of the supraspinatus, but now with 5/5 strength and near the full active range of motion, and negative Empty can, Hawkins, and Neer tests. The patient at this time had not yet started physical therapy. We recommended the patient begin a short course of physical therapy in hopes that she would see further improvement and potential complete resolution of her injury. We recommended follow up in eight weeks for reevaluation, with planned repeat radiographs to assess the level of calcific burden.

**Figure 3:**
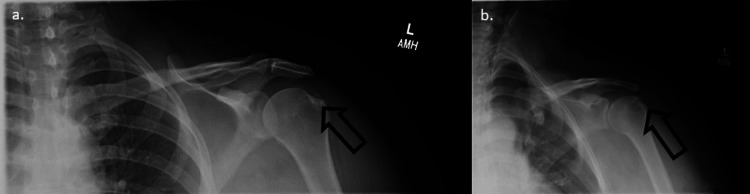
Radiographs of the left shoulder at eight week follow up demonstrating significant regression of calcific deposits. a) Anterior-posterior view. b) Internal rotation view.

At her eight-week follow-up, the patient was very pleased with her progress. She still had residual pain at the end of passive and active range of motion but had much less difficulty with daily activities. The patient at this time had not yet started physical therapy. Physical exam showed mild tenderness with palpation of the supraspinatus tendon but showed no pain with resistance testing or special testing. We obtained a repeat radiograph of the shoulder that confirmed significant regression of calcium deposits (Figure 3). We recommended the patient begin physical therapy so she could see even further improvement in her symptoms. We talked about the potential for a repeat ultrasound-guided methylprednisolone injection if symptoms persisted or returned.

The patient came in for follow-up after another eight weeks. The patient now reported 100% improvement in her symptoms. She had completed six weeks of physical therapy and had transitioned to a pure home exercise program. Physical exam showed no tenderness with palpation of the supraspinatus tendon, and no pain with passive or active range of motion, resistance, or special testing. Patient was instructed to continue her at-home exercise program and schedule a follow-up if symptoms returned. A repeat radiograph was not indicated due to the full resolution of symptoms.

## Discussion

The lifetime prevalence of shoulder pain is estimated to be as high as 67% in some reports, with rotator cuff pathology being the most common cause [1]. A well-known culprit of rotator cuff pathology is RCCT, with deposits found in 7-17% of patients with shoulder pain [3]. Despite the common occurrence of RCCT, there are currently no high-quality evidence-based guidelines for its treatment [3]. As mentioned, management typically begins with conservative measures such as physical therapy, NSAIDs, and corticosteroid injections. Cases refractory to these methods are candidates for other nonsurgical methods of management such as ESWT or ultrasound-guided barbotage. Surgical intervention is required for the most refractory cases. The available surgical techniques (arthroscopic bursectomy, decompression with acromioplasty, open calcium deposit removal) have been shown to have satisfactory clinical outcomes [3].

In this case report, the patient underwent ultrasound-guided glucocorticoid injection targeting on and around the calcium deposits along the supraspinatus tendon. Despite a lack of adherence to physical therapy recommendations, the patient still showed improvement at both four-week and eight-week follow-up appointments. Improvement was indicated by decreased tenderness to palpation, decreased pain with ROM, and patient satisfaction at both visits. A radiograph of the left shoulder at the eight-week appointment further corroborated these findings and demonstrated reduced calcific burden. This case exemplifies how ultrasound-guided injection is an efficacious next step for RCCT that is refractory to NSAIDs and physical therapy without jumping to surgical interventions.

Despite the lack of clinical guidelines for management of RCCT, many studies have investigated other second line options. ESWT, a method of using acoustic waves to dissolve calcium deposits, has shown mixed results. One study showed that it is effective in calcium deposit resolution, showing complete resolution of deposits in 55.6% of patients [6]. Another study demonstrated a lack of clinically significant improvement in pain and function when compared to placebo [5]. Beyond clinical efficacy, ESWT requires an experienced therapist and equipment that is highly specialized and expensive [6]. Ultrasound-guided barbotage, a method of using a needle to break up calcium deposits, has been shown to have improved pain scores and resolution of calcium deposits when compared to ESWT [6,7,8]. However, it has associated risk of damage to the rotator cuff tendons, requires even more specialized personnel, and can be very painful for the patient [6,9]. Compared to surgical intervention, ultrasound-guided injection limits the need for advanced imaging, such as MRI. Its use also eliminates the risk of postsurgical complications such as adhesive capsulitis or prolonged shoulder stiffness, which have been shown to occur in 1-12% of patients [10,11,12]. As a second-line treatment, ultrasound-guided glucocorticoid injection could settle uncertainties in RCCT management. While there are no comparative studies evaluating landmark or palpation-guided injection vs ultrasound-guided injection, ultrasound has the benefit of direct visualization of calcium deposits, resulting in a more precise steroid injection. Further investigation into this method is needed to confirm its role in RCCT treatment, but this case is a first step in establishing its efficacy.

## Conclusions

This case study demonstrates the efficacy of ultrasound-guided glucocorticoid injections for the treatment of RCCT. Our patient achieved near 100% remission with the ultrasound-guided injection alone, and 100% remission after supplementing treatment with physical therapy. Although conservative treatment with NSAIDs and physical therapy should serve as the first step in management of RCCT, ultrasound-guided glucocorticoid injections around the site of calcific deposits is an option that can provide quick remission with minimal risk.
